# Fe–Zn alloy, a new biodegradable material capable of reducing ROS and inhibiting oxidative stress

**DOI:** 10.1093/rb/rbae002

**Published:** 2024-01-12

**Authors:** Shuaikang Yang, Weiqiang Wang, Yanan Xu, Yonghui Yuan, Shengzhi Hao

**Affiliations:** School of Materials Science and Engineering, Dalian University of Technology, Dalian 116024, PR China; School of Materials Science and Engineering, Dalian University of Technology, Dalian 116024, PR China; School of Materials Science and Engineering, Dalian University of Technology, Dalian 116024, PR China; Clinical Research Center for Malignant Tumor of Liaoning Province, Cancer Hospital of Dalian University of Technology, Shenyang 110042, PR China; School of Materials Science and Engineering, Dalian University of Technology, Dalian 116024, PR China

**Keywords:** biodegradable iron, Fe–Zn alloys, reactive oxygen species, oxidative stress, cytocompatibility

## Abstract

Fe-based biodegradable materials have attracted significant attention due to their exceptional mechanical properties and favorable biocompatibility. Currently, research on Fe-based materials mainly focuses on regulating the degradation rate. However, excessive release of Fe ions during material degradation will induce the generation of reactive oxygen species (ROS), leading to oxidative stress and ferroptosis. Therefore, the control of ROS release and the improvement of biocompatibility for Fe-based materials are very important. In this study, new Fe–Zn alloys were prepared by electrodeposition with the intention of using Zn as an antioxidant to reduce oxidative damage during alloy degradation. Initially, the impact of three potential degradation ions (Fe^2+^, Fe^3+^, Zn^2+^) from the Fe–Zn alloy on human endothelial cell (EC) activity and migration ability was investigated. Subsequently, cell adhesion, cell activity, ROS production and DNA damage were assessed at various locations surrounding the alloy. Finally, the influence of different concentrations of Zn^2+^ in the medium on cell viability and ROS production was evaluated. High levels of ROS exhibited evident toxic effects on ECs and promoted DNA damage. As an antioxidant, Zn^2+^ effectively reduced ROS production around Fe and improved the cell viability on its surface at a concentration of 0.04 mmol/l. These findings demonstrate that Fe–Zn alloy can attenuate the ROS generated from Fe degradation thereby enhancing cytocompatibility.

## Graphical Abstract

**Figure:**
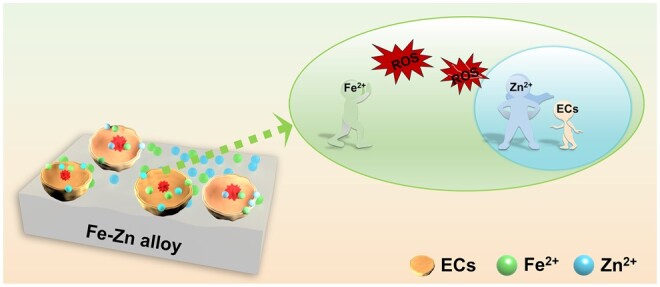

## Introduction

Fe-based alloys, considered one of the most potential candidates for biodegradable implanted materials, have attracted more attention at present. Research on their biosafety, mechanical properties and biodegradation properties [1–3] is a hot topic. However, slow corrosion rates have hindered the development of Fe-based materials [4–7]. To this day, various alloying [8–10], surface modification [11–13] and advanced manufacturing [14, 15] methods have been used to regulate the degradation behavior of Fe-based alloys. In our previous work, we successfully prepared a new Fe–Zn alloy through electrodeposition. *In vitro* tests demonstrated improved corrosion performance compared to pure Fe, and the Fe–Zn alloys with low Zn content exhibited satisfactory mechanical properties [16]. Based on these findings, further exploration of its biocompatibility is warranted.

Fe (iron), an essential trace element for sustaining life, plays a crucial role in biosynthesis and metabolism. It is responsible for maintaining the enzyme activity in mammalian cells and regulating cell proliferation and death [17]. However, due to its redox-active nature, it is necessary to maintain iron homeostasis within cells. Fe overload will generate excessive reactive oxygen free radicals through the Fenton reaction, promoting oxidative stress that can damage DNA, protein or lipid resulting in cell apoptosis or necrosis [18]. Schieber *et al.* state that reactive oxygen species (ROS) have two faces: redox biology and oxidative stress, exerting influences on both physiological and pathological conditions. Redox biology refers to a slight elevation in ROS levels, which triggers signaling pathways to initiate biological processes. This phenomenon proves advantageous for stem cell regeneration, cell proliferation and differentiation, thereby promoting robust immune responses and even contributing to longevity. Oxidative stress means high levels of ROS that can induce damage to DNA, protein or lipid, leading to histopathological impairments [19, 20]. It assumes a pivotal role in the pathogenesis of diverse chronic human ailments, encompassing atherosclerosis and associated vascular disorders, mutagenesis and oncogenesis, neurodegeneration, immunological dysfunctions, as well as the process of aging. Additionally, excessive intracellular ROS is closely associated with ferroptosis—a non-apoptotic cell death mechanism driven by lipid peroxidation proposed by Dixon et al. [21]. The regulation of ferroptosis occurs through specific pathways and encompasses various biological contexts. Triggers for ferroptosis include Fe overload, Glu (glutamate), SLC7A11 (solute carrier family 7 member 11) suppression, GPX4 (glutathione peroxidase 4) depletion and PUFA (polyunsaturated fatty acid) uptake [22]. The occurrence and regulation mechanisms of ferroptosis are being extensively investigated, attracting the attention of numerous researchers. However, it is undeniable that ROS generated by Fe overload plays a crucial role in promoting ferroptosis. Therefore, concerning oxidative stress or ferroptosis, careful consideration should be given to the ROS levels resulting from the degradation of Fe-based implant materials, especially those with a rapid degradation rate.

Currently, numerous animal and clinical trials have extensively investigated the efficacy of antioxidants, including sulfur and selenium antioxidants [23], iridium complexes [24] and zinc (Zn) [25], in mitigating oxidative stress. Notably, among these antioxidants, Zn holds a distinctive status as a redox-inert metal. In accordance with the definition of biodegradable metals [26], magnesium [27, 28] and zinc [29] stand out as biodegradable metal materials distinct from iron. The incorporation of zinc into iron-based materials may concurrently confer antioxidant properties and expedite material degradation. Zn is the second most abundant trace element in biology and is widely acknowledged as an essential micronutrient for all organisms [30–32]. The impact of Zn on cell activity is bidirectional, with high concentrations being cytotoxic, while low concentrations have no adverse effects on cell viability [33]. Instead, lower levels of Zn promote crucial cellular processes such as adhesion, proliferation, migration and DNA damage repair and enhance the expression of F-actin and adhesion protein. Numerous studies have verified that Zn, as an antioxidant, reduces ROS production and protects proteins from free radicals, showing an outstanding performance in antioxidative stress [34–36].

So, investigating the potential synergistic effect of Zn and Fe in reducing the oxidative stress caused by Fe is highly appealing. In this study, we investigated the effects of Fe^2+^, Fe^3+^ and Zn^2+^ on the activity of human endothelial cells (ECs) and wound healing ability. We investigated the toxic effects of high levels of ROS on ECs by assessing the ROS production and cell viability around Fe and Fe–Zn alloys. Moreover, we evaluated the influence of ROS around the alloys on DNA damage within the nucleus. To further confirm whether Zn ions can inhibit ROS production and cytotoxicity caused by Fe overload, we also explored the cell activity and ROS production of pure Fe in the medium environment with different zinc ion concentrations.

## Materials and methods

### Material preparation

**Figure 1.**
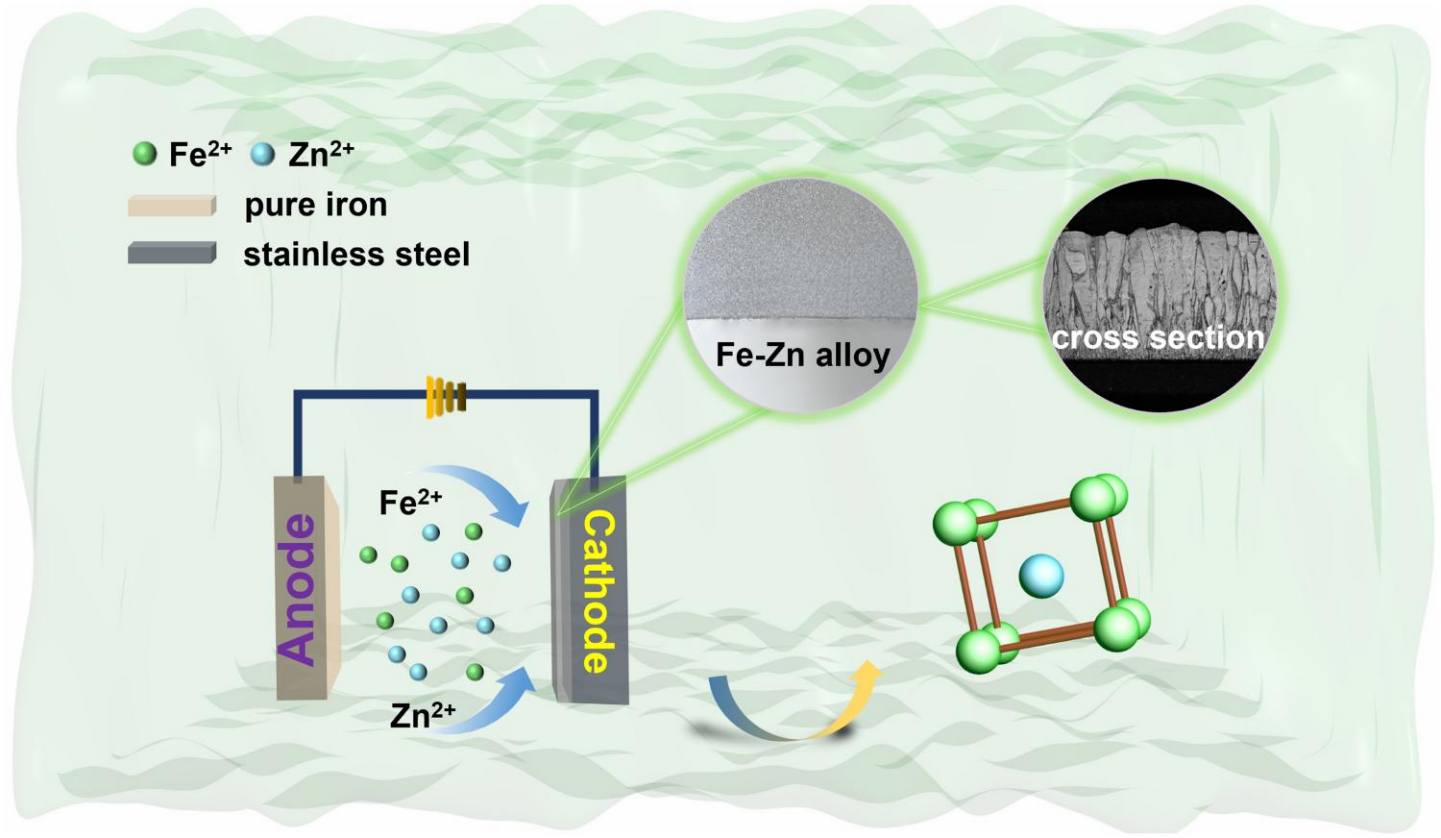
Schematic diagram of the preparation of Fe–Zn alloys.

Fe–Zn alloys were fabricated via pulse electrodeposition (Figure 1), based on our previous research [16]. The specific electrolyte composition is presented in Supplementary Table S1. The stainless less sheet and commercial pure Fe plate were used as a working cathode and anode, respectively. After that, the alloys were annealed at a temperature of 450°C for a duration of 20 min in an argon atmosphere. The coating layers were carefully removed from the substrate using mechanical methods. All the alloys with a deposited thickness of 80 μm were cut into small 1 × 1 cm^2^ squares. Then they underwent thorough cleaning and sterilization through ultraviolet irradiation for a duration of 30 min.

### Microstructure and composition characterization

For this analysis, the samples underwent initial sealing with resin. Subsequently, four types of water abrasive sandpaper (600#, 1000#, 1500# and 2000#) were used to grind the samples successively, and finally, they were polished with 1.5-µm diamond polishing paste. When observing the microstructure, a 25 vol.% solution of nitric acid in alcohol was employed for etching purposes lasting 5 s. Field emission scanning electron microscopy (FE-SEM, IT800-SHL, JEOL, Japan) and field emission electron probe microanalyzer (EPMA, JXA-8530F PLUS, JEOL, Japan) were used to analyze the cross-sectional of the alloys. The composition results were averaged over five random points.

### Preparation of ionic solutions

Zinc chloride (ZnCl_2_, Aladdin, China), ferrous chloride (FeCl_2_, Aladdin, China) and ferric chloride (FeCl_3_, Aladdin, China) compounds were dissolved in deionized water to prepare 80 mmol/l stock solutions. Then the stock solutions were serially diluted by endothelial cell medium (ECM) without fetal bovine serum (FBS, ScienCell, USA) and EC growth factor (ECGF, ScienCell, USA) supplementation to 0.01, 0.02, 0.04, 0.08, 0.1, 0.2, 0.5, 1, 2, 3, 4 and 5 mmol/l and the ECM without FBS and ECGF acted as a control group. Because Fe^2+^ is unstable in solution and easily oxidized to Fe^3+^, ionic solutions were prepared when used.

### Cytocompatibility test

Human umbilical vein ECs were cultured with a complete medium (including 5% FBS, 1% penicillin-streptomycin mixture (Sciencell, USA) and 1% ECGF) at 37°C and 5% CO_2_.

To investigate the cell compatibility of the samples, comprehensive cytotoxicity and cell migration experiments were conducted using ion solutions, while adhesion and proliferation experiments were performed on alloy samples. Detailed descriptions of these experiments can be found in the Supplementary data.

### Assay for ROS generation, cell death staining and DNA damage

The sterilized alloys were placed in six-well plates. Each well was then supplemented with 3 ml of cell suspension with a concentration of 5 × 10^4^ cells/ml. After being cultured for 24 and 48 h, Fe and Fe–Zn alloys were removed from the wells. The production of intracellular ROS was detected using the DCFH-DA probe of the ROS assay kit (Beyotime Biotechnology, China). ECs were stained with 0.5 μmol/l DCFH2-DA for 15 min in darkness. Then, the staining solution was removed, and the wells were washed twice with PBS. Finally, the fluorescence microscope was used to observe and take photos.

To detect live and dead cells, fluorescence staining was performed on the cells using a cell viability/cytotoxicity assay kit (Beyotime Biotechnology, China). The photos were taken using the fluorescence microscope.

DNA damage assay kit (Beyotime Biotechnology, China) was selected to detect the DNA damage marker γ-H2AX (phosphorylated H2AX) by immunofluorescence staining. After staining, the γ-H2AX showed green fluorescence. The nucleus was stained into blue fluorescence with a DAPI staining solution provided by the kit. When the cells are cultured under normal conditions, the content of γ-H2AX is very low, and there is almost no green fluorescence in the nucleus. The damage degree of cells can be judged by detecting the intensity of green fluorescence in the nucleus under different stimulation conditions. Finally, the fluorescence microscope was used to observe and take photos.

### Cytotoxicity on the surface of pure iron by Zn ion stimulation

The sterilized pure iron was placed in 24-well plates. The cell suspension with different Zn ion concentrations was cultured with pure iron at a density of 5 × 10^4^ cells/ml and 1 ml per well. To measure the cell viability of the cells on the pure iron, the pure iron was moved into new wells after incubation for 72 h and then washed with PBS twice. The culture medium containing 10% CCK-8 was added and cultured for 2 h. The complete medium containing CCK-8 and cells was used as a positive control, and the complete medium containing CCK-8 but without cells acted as a negative control. The supernatant was transferred into 96-well plates to test their absorbance.

### Statistical analysis

The quantitative experiments in *in vitro* biological evaluation were conducted with a minimum of three repetitions, and univariate analysis of variance was performed for all quantitative experimental results. The difference is considered statistically significant when the *P* value is <0.05, and it is deemed extremely significant if the *P* value is <0.01. The qualitative experiments on biological evaluation were also repeated at least three times to ensure the consistency of the results.

## Results

### Microstructure and composition of Fe–Zn alloys

**Figure 2.**
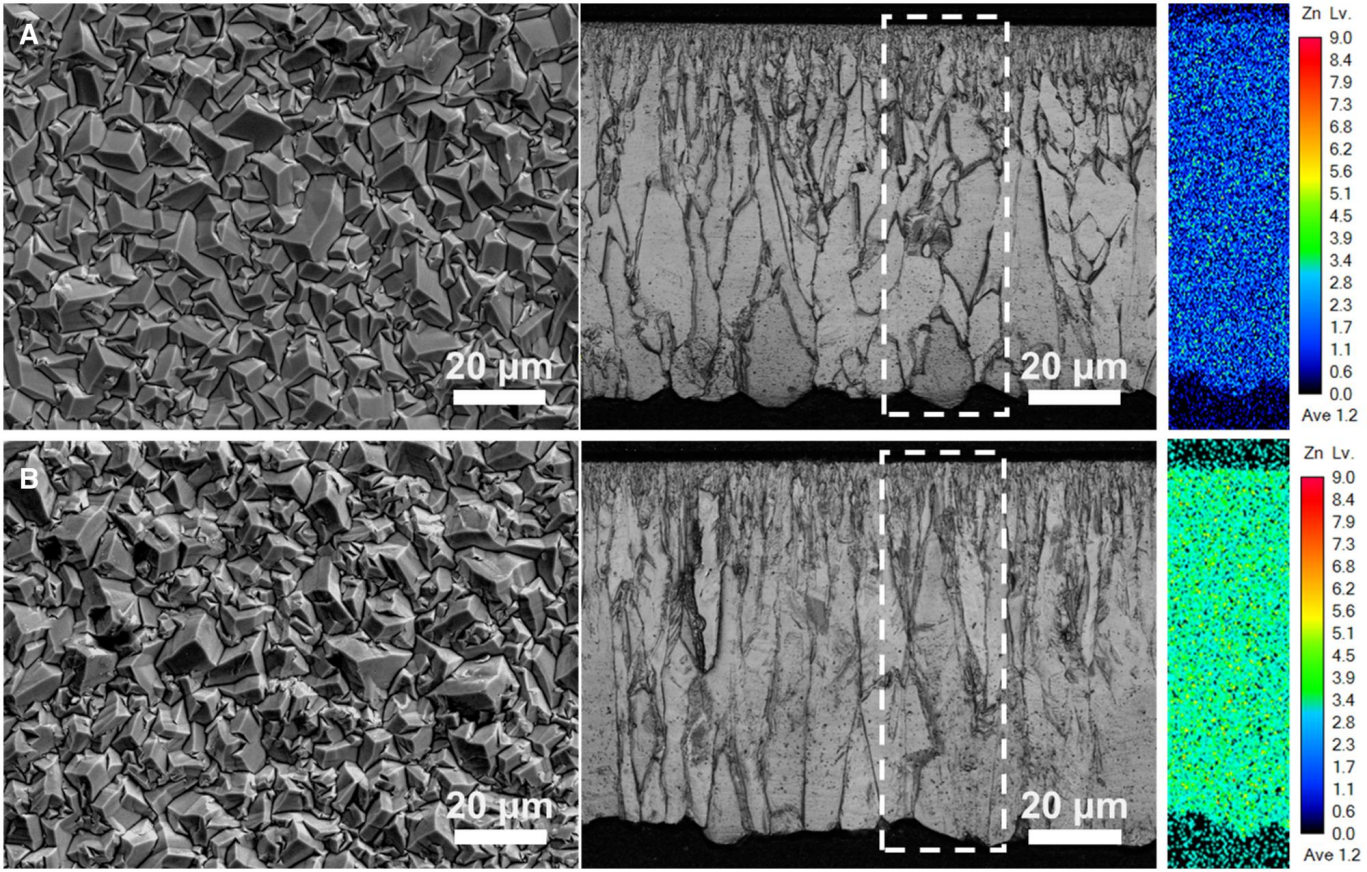
FESEM micrographs, cross-sectional images and zinc distribution map of the (**A**) Fe–2.1Zn and (**B**) Fe–4.6Zn.

The microstructure and composition of alloys were controlled by adjusting the pulse frequency (100 Hz for Fe–4.6Zn, 1000 Hz for Fe–2.1Zn) with a constant current density at 10 A/dm^2^ (Table 1). The obtained alloys exhibited a columnar crystal microstructure, with a uniform distribution of Zn throughout the matrix (Figure 2). The solubility of Zn in Fe at 300°C is approximately 2%, as indicated by the Fe–Zn phase diagram [37], which drops even lower at room temperature. The XRD results of Fe–Zn alloys are presented in Supplementary Figure S1. Both Fe–2.1Zn and Fe–4.6Zn exhibited a single-phase structure of α-Fe, indicating that the electrodeposited Fe–Zn alloys are supersaturated solid solutions.

**Table 1.** Composition of Fe–2.1Zn and Fe–4.6Zn

Alloys	Fe (wt.%)	Zn (wt.%)	O (wt.%)
Fe–4.6Zn	95.3 ± 0.1	4.6 ± 0.1	0.1 ± 0.1
Fe–2.1Zn	92.7 ± 0.1	2.1 ± 0.1	0.1 ± 0.1

### Cytocompatibility and cell migration in ionic solutions

**Figure 3.**
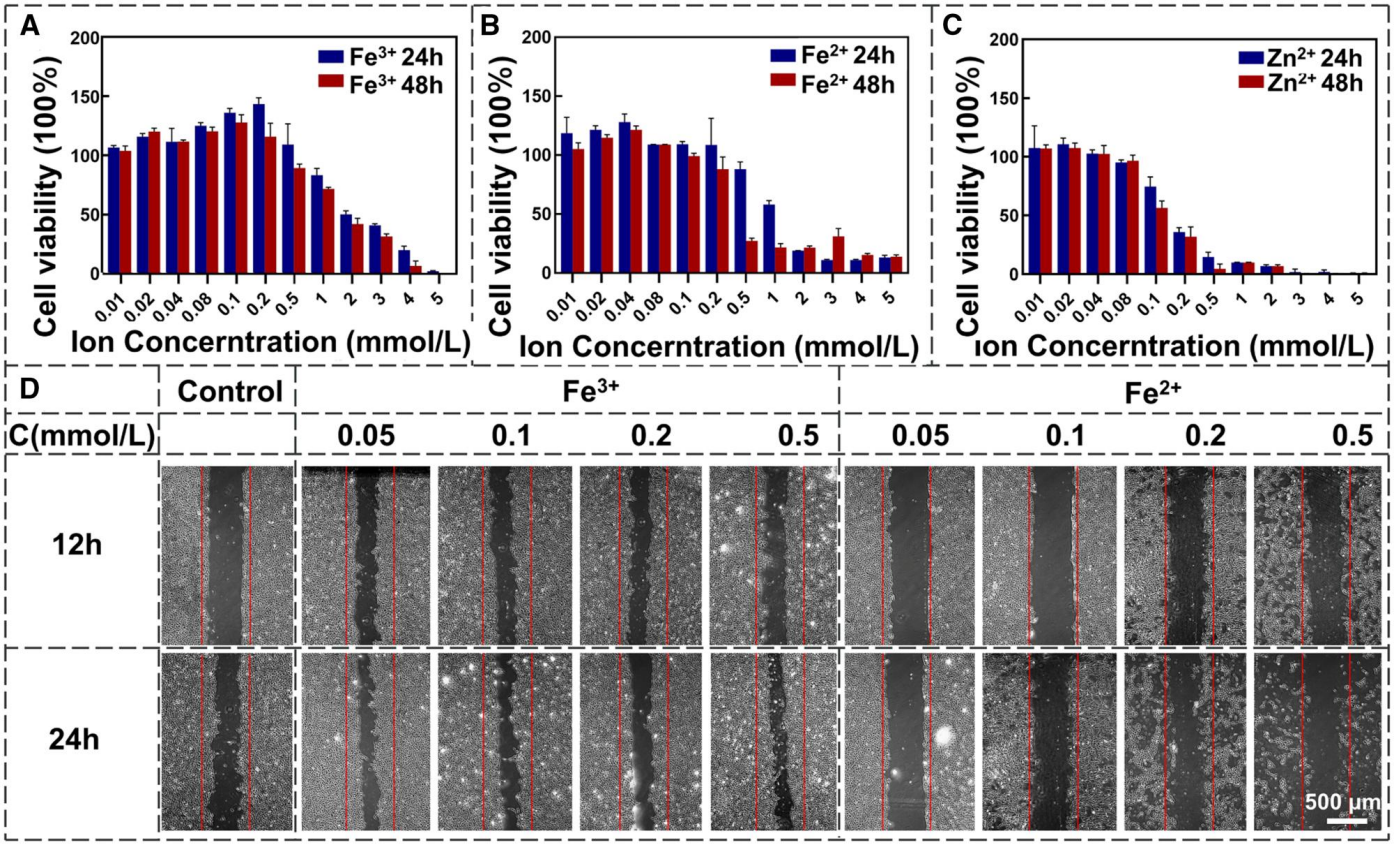
The effects of varying concentrations of Fe^3+^ (**A**), Fe^2+^ (**B**) and Zn^2+^ (**C**) in the culture medium on cell viability and the visualization of cell migration under different ion concentrations of Fe^3+^ and Fe^2+^ (**D**).

The degradation of Fe–Zn alloy will generate the ions of Fe^2+^, Fe^3+^ and Zn^2+^, which have distinct physiological roles in the human body. However, it is challenging to differentiate the specific contributions of these ions when directly studying the Fe–Zn alloy. To understand the effect of individual ions on cytocompatibility, artificially prepared ion solutions with varying concentrations were initially evaluated for cell viability and migration ability (Figure 3). It was noted that the FBS may inhibit the generation of ROS [38]. To eliminate any potential interference, the ECM without FBS and the ECGF was used to culture cells. ECs exhibited greater tolerance to Fe^2+^ and Fe^3+^ than Zn^2+^. The IC50 value, representing a substance concentration required to induce a 50% reduction in cellular response compared to untreated cells [39], for Zn^2+^ ions is about 0.1 mmol/l, while those for Fe^2+^ and Fe^3+^ were around 0.5 and 2 mmol/l, respectively (Figures 3A–C). The inhibitory effect of divalent iron ions on cell activity seems greater. Some researchers have highlighted the significance of iron’s oxidation state in its capacity to contribute to ferroptosis [40]. Fe^3+^ is typically inert and stored within ferritin, except in the active site of lipoxygenases, where Fe^3+^ assumes an active role as the enzyme. Fe^2+^, however, is active. It can be oxidized to Fe^3+^ ions, as is accompanied by the generation of ROS. The capacity of Fe^2+^ to induce ROS production may explain why it has a higher inhibitory effect on cell activity. Previous studies also showed that the metabolic activity of ECs decreases sharply when iron ion concentrations exceed 0.9 mmol/l, regardless of the incubation time [41]. Notably, the iron ion solution mentioned in the study was a material extract containing both Fe^2+^ and Fe^3+^ ions, and its concentration limit falls within the range of IC50 values of the two iron ions measured in this study. Figure 3D shows the effects of three ions on cell migration ability. At the concentration range of 0.05–0.5 mmol/l, Fe^3+^ exhibited a stimulatory effect on cell migration compared with the control group, whereas Fe^2+^ had an inhibitory effect on cell migration. Additionally, Fe^2+^ seemingly reduced cell adhesion at a concentration range of about 0.2–0.5 mmol/l.

### Cytocompatibility assessments of Fe and Fe–Zn alloys

**Figure 4.**
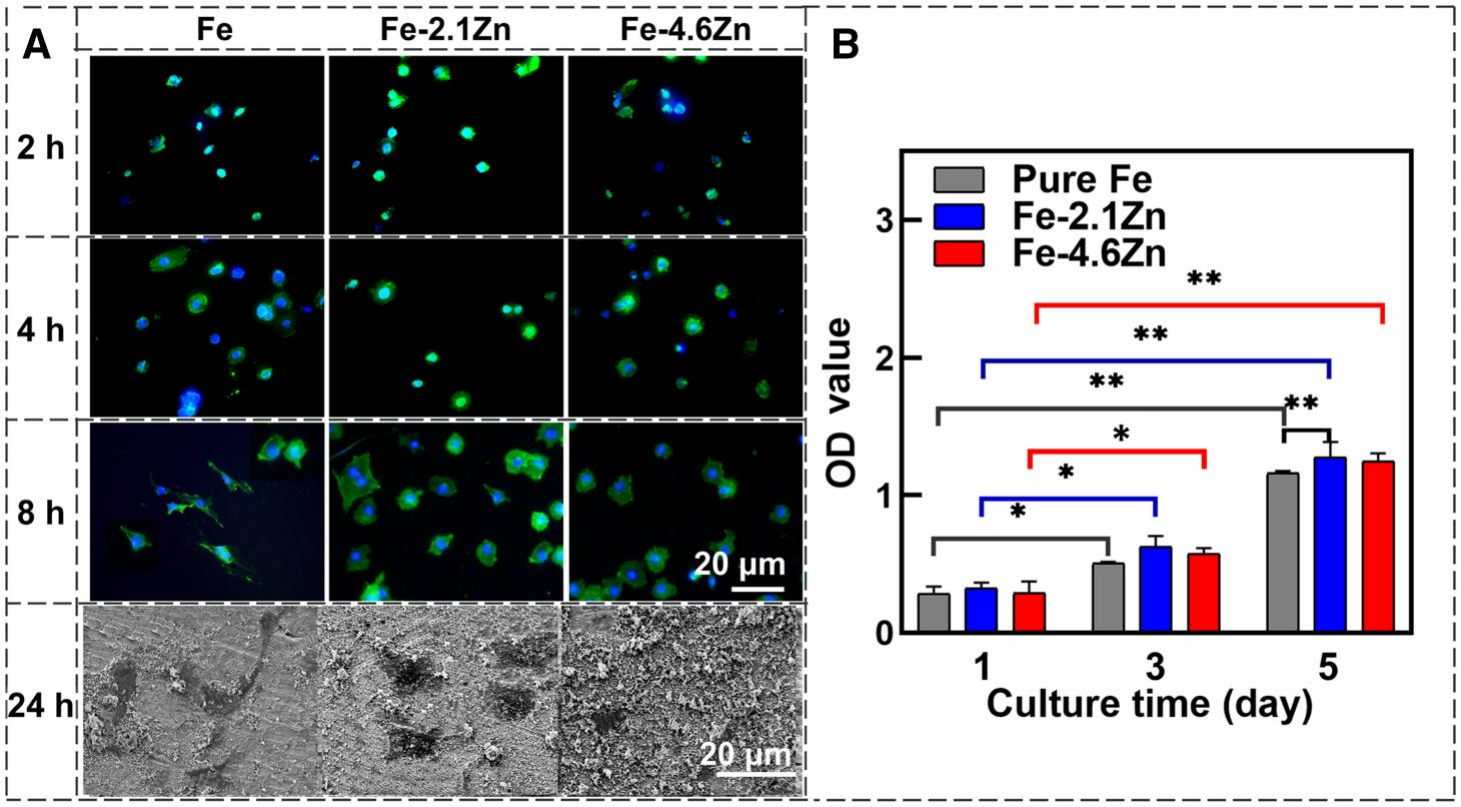
The ECs adhesion morphology (**A**) and cell proliferation (**B**) on the surface of pure Fe and Fe–Zn alloys. Cytoplasm was stained with Actin-Tracker green-488 (green). Cell nuclei were stained with DAP (blue). *P < 0.05, **P < 0.01.

Figure 4A depicts the adhesion morphology of ECs on the alloy surface. No significant difference was observed in the morphology of ECs on all samples after 2- and 4-h incubation. After incubation for 8 and 24 h, ECs exhibited better spreading morphology on pure Fe surface compared to Fe–Zn alloys. The corrosion rates of Fe–Zn alloys, especially Fe–4.6Zn, were higher than that of pure Fe (Supplementary Figure S2), resulting in more corrosion products accumulating on their surfaces (Supplementary Figure S3), which may hinder cell adhesion and spreading [42]. Figure 4B describes the proliferation of cells on three sample surfaces. Although the cells did not spread well on the Fe–Zn alloy surface in the early stages of culture, the cell proliferation on all sample surfaces was comparable after 1 day of culture. After 3 days of cell culture, there was a significant increase in solution absorbance of the solution, indicating normal cell proliferation. Notably, after 5 days of culture, the cell activity observed on the Fe–2.1Zn alloy surpassed that of pure Fe and exhibited a statistically significant difference (*P* < 0.01). These findings suggest that despite faster corrosion rates compared to pure Fe, the presence of Zn in Fe–Zn alloys enhances cellular activity, which may be attributed to the antioxidative properties of Zn.

### The effects of Zn on ROS production, cell activity and DNA damage of Fe–Zn alloy

**Figure 5.**
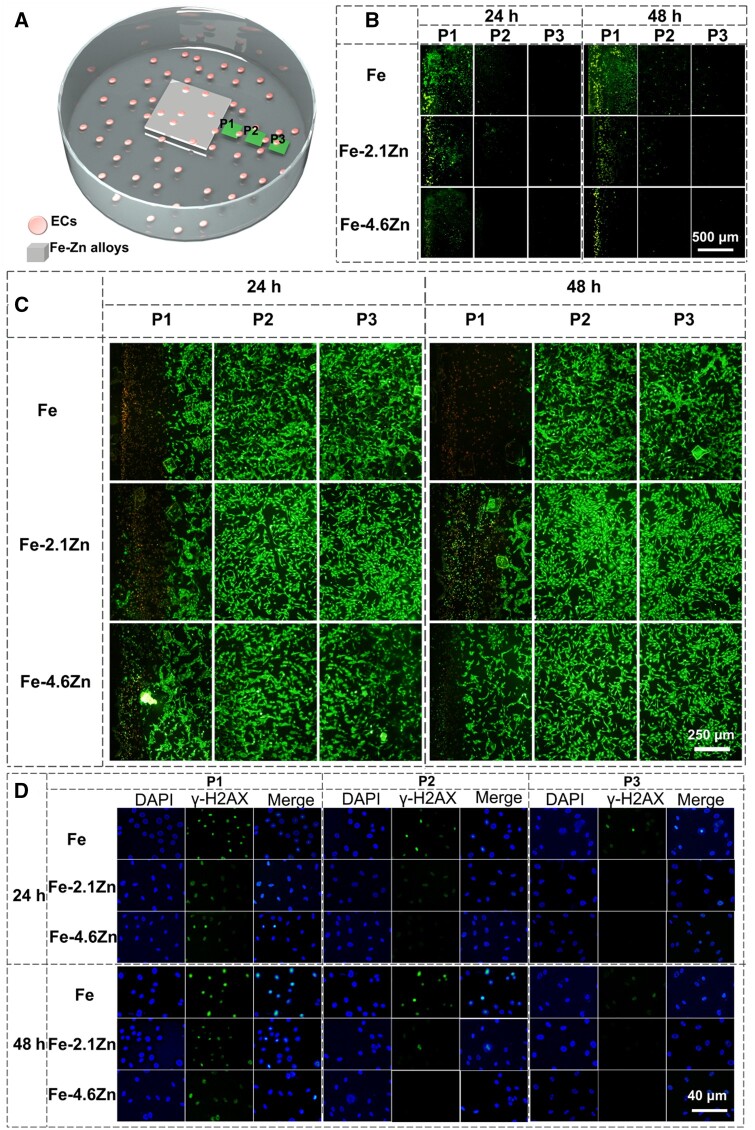
Effects of different positions around alloy samples on ROS production, cell viability and DMA damage. (**A**) Schematic diagram of P1, P2 and P3 positions around the alloy (**B**–**D**) are ROS production, cell viability (living cell/green, dead cell/red) and DNA damage, respectively, at P1, P2 and P3 positions. DAPI (blue) was used for the identification of cell nuclei and γ-H2AX fluorescence (green) for DNA damage.

While ROS is primarily generated through the corrosion process [43] of Fe-based materials, it should be noted that excessive soluble Fe^3+^ can also contribute to ROS production. Therefore, considering the dimensions of the alloy sample utilized, three specific locations within the region where soluble products are released from the sample were selected for subsequent detection. As depicted in Figure 5A, the alloy was placed in a six-well plate, and three equidistant positions surrounding the alloy sample were designated as P1, P2 and P3, representing the nearest neighbor, next neighbor and far neighbor positions, respectively. In this study, ROS production in cells was detected using the DCFH2-DA probe (see Supplementary data). The green fluorescence intensity in cells directly reflects the ROS levels. Due to its short half-life, ROS is generated locally during the reaction and gradually attenuated as it diffuses over distance. Consequently, there were significant differences in the ROS production at P1, P2 and P3 around the alloys shown in Figure 5B, with closer proximity to the alloy resulting in higher levels of ROS production. Moreover, distinct variations were observed regarding the ROS production around different samples. Pure Fe exhibited greater ROS production compared to Fe–Zn alloys; furthermore, even at position P3 visible fluorescence indicating high levels of ROS was still present for pure Fe. As Zn content increased within Fe–Zn alloys, the amount of produced ROS gradually decreased. Fe–4.6Zn alloy only displayed weak ROS fluorescence at site P1 after 24- and 48-h incubation. Figure 5C shows cell viability at P1, P2 and P3 around the alloy. It can be seen that cell viability around the alloy corresponded to its ROS production; specifically, higher ROS production led to increased cell death (indicated by red fluorescence). The number and area of dead cells around Fe were greater than those surrounding Fe–Zn alloys. Figure 5D demonstrates the impact of ROS on DNA damage. DNA damage was observed at the P1 sites for all alloys (as evidenced by stronger γ-H2AX fluorescence) and it was weakened far away from samples. DNA damage around pure Fe was greater than that around Fe–Zn alloys, and at the P3 site, such damage was detected only for the pure Fe sample. The following point deserves attention: ROS production is an inevitable by-product of cellular aerobic respiration. A small amount of ROS does not affect cellular activity; only excessive ROS can result in cell death and DNA damage. Furthermore, there is no direct correlation between DNA damage and cell death. For example, the P2 position of pure Fe showed signs of DNA damage, but few dead cells were found.

### The effect of Zn on Fe-induced oxidative damage in cells

**Figure 6.**
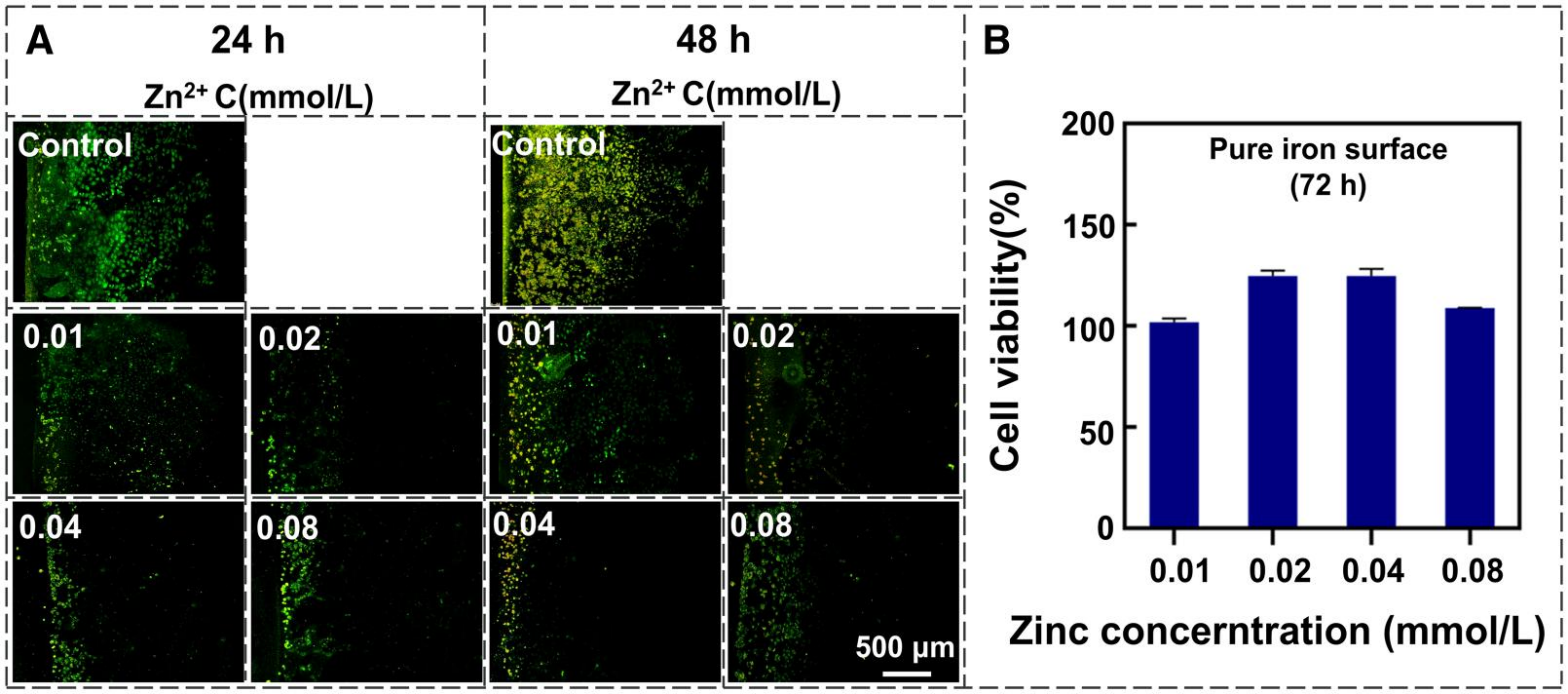
ROS Production around pure iron after 24 and 48 h of cell culture (**A**) and cell viability on pure iron surface after 72 h of cell culture (**B**).

To investigate the independent effect of Zn in mitigating the cell damage caused by Fe overload, a culture medium with varying concentrations of Zn ions was added to wells containing cells and pure iron samples. The Zn ion concentrations in the medium were 0.01, 0.02, 0.04 and 0.08 mmol/l. The upper limit of Zn ion concentration was determined based on the findings from Figure 3C, which shows that ECs were not inhibited when the Zn ion concentration was below 0.08 mmol/l. Figure 6A shows the ROS production in the adjacent area around the pure iron samples in the medium with different Zn^2+^ concentrations. Compared with the control group without Zn ions, the ROS production around the pure iron decreased gradually with the increase of Zn^2+^ content and reached the lowest at 0.04 mmol/l. When the Zn^2+^ concentration increased to 0.08 mmol/l, no significant change in ROS levels was observed in cells around pure iron, which may be attributed to the excess of Zn^2+^ that also induces oxidative damage in cells [44]. As the culture time extended from 24 to 48 h, there was no observed increase in ROS production across all experimental groups. Subsequently, the cells were cultured for an additional 24 h, and cell viability on the pure iron surface was assessed, with results presented in Figure 6B. The cell viability increased significantly when the Zn^2+^ concentration was in the range of 0.02–0.04 mmol/l, reaching its peak at 0.04 mmol/l. The results depicted in Figure 6 demonstrate a positive correlation between the reduction of ROS levels in the cells around the pure iron sample and an enhancement in cell viability on its surface, confirming that Zn ions can alleviate the ROS accumulation in cells and reduce the inhibitory effect of ROS on cell activity within the range of <0.08 mmol/l.

## Discussion

**Figure 7.**
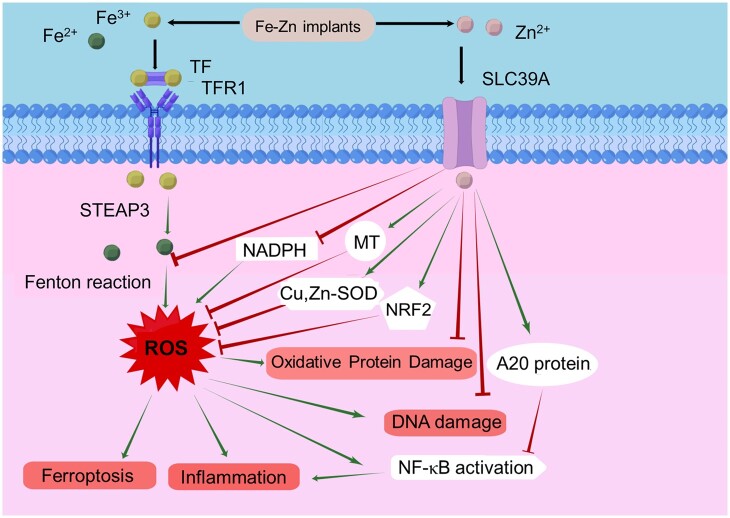
Schematic diagram of Zn as an antioxidant. A20, zinc-finger protein A20; MT, metallothionein; NADPH, nicotinamide adenine dinucleotide phosphate; NF-κB, nuclear factor kappa-B; NRF2, nuclear factor, erythroid derived 2, like-2); SLC39A, solute carrier family 39; STEAP3, six-transmembrane epithelial antigen of prostate 3; TF, transferrin; TFR1, transferrin receptor protein 1.

The degradation of Fe or Fe-based alloy implants will produce Fe^2+^ and Fe^3+^ ions. Part of Fe^3+^ ions enter cells through the TF/TFR1 transport system and transform into Fe^2+^ under the action of STEAP 3 (Figure 7). Then they selectively form various complexes with substances having iron-binding properties, thereby participating in a series of physiological and biochemical reactions. As the concentration of these complexes approaches saturation, Fe^2+^ irons will accumulate within cells, forming an unstable pool of iron, and participate in the Fenton reaction to produce ROS [21]. Excessive ROS will induce oxidative stress and ferroptosis.

The intracellular signaling molecule Zn participates in a multitude of cellular mechanisms and gains entry into the cell through SLC39A [45]. Zn can be used as an antioxidant to reduce the production of ROS in cells. Zinc exerts its antioxidant function through two distinct mechanisms: (i) direct binding of zinc to the sulfhydryl groups and (ii) seizing the binding sites of oxidizing metals (such as iron), thereby attenuating their electron transfer capacity in specific environments and exerting precise control over the process of protein oxidation [46]. The direct binding of Zn and sulfhydryl groups can protect various proteins containing sulfhydryl groups from free radical attack and prevent ROS damage [47]. The element Zn has the ability to protect ECs from hydrogen peroxide by stimulating glutathione biosynthesis through NRF2-dependent mechanisms [48]. Furthermore, the antagonism of Zn to Fe prevents the excessive accumulation of Fe ions in the cell and reduces its capacity to catalyze the Fenton reaction and facilitate the generation of hydroxyl radicals.

Zn as an antioxidant, can also reduce the formation of free radicals or tissue damage caused by free radicals in several other ways [49]. Zn serves as an inhibitor of NADPH oxidase [50], a stimulator of metallothionein [51] (effective scavenger of radicals), and is an essential component of Cu, Zn-SOD [52]. Additionally, Zn enhances the production of a zinc-finger protein called A20, which upregulates NF-kB activation via the TRAF pathway and subsequently reduces inflammatory cytokine production [53]. Therefore, the presence of zinc results in the manifestation of both antioxidant and anti-inflammatory properties (Figure 7).

The Fe–Zn alloys prepared by pulsed electrodeposition showed a single-phase solid solution structure of α-Fe (Supplementary Figure S1). The cross-sectional elemental distribution analysis revealed a uniform dispersion of Zn throughout the alloy matrix (Figure 2). The long-term immersion tests demonstrated that the corrosion products consistently maintained a constant iron-zinc ratio (Supplementary Figure S3). These findings highlight the ability of the Fe–Zn alloy to continuously release Zn ions, making it an ideal solution for addressing issues related to Fe overload, excessive ROS production, and oxidative stress caused by Fe-based implant materials.

## Conclusions

In this study, the effects of Fe^2+^, Fe^3+^ and Zn^2+^ ions, which are potentially derived from the degradation of Fe–Zn alloys, on the activity of ECs were investigated *in vitro*. Notably, Fe^3+^ and Fe^2+^ exhibited higher tolerance toward ECs, with IC50 values of 0.5 and 2 mmol/l, respectively. In contrast, the tolerance of Zn^2+^ was the lowest, characterized by an IC50 of 0.1 mmol/l. Among the two Fe ions, Fe^2+^ participated in the Fenton reaction, which produced excessive reactive oxygen radicals and reduced cell activity. Fe^3+^, possessing a more stable chemical state, enhanced cell activity and promoted cell migration at low concentrations. In the evaluation of direct cell culture, the pure Fe, Fe–2.1Zn and Fe–4.6Zn alloys were examined. Among these, the Fe–4.6Zn alloy, characterized by its rapid corrosion rate, produced more corrosion products during culture. These products hindered cell adhesion and spreading, but normal cell proliferation was observed on the surfaces of all three metals. Meanwhile, varying levels of ROS release were detected around Fe and Fe–Zn alloys, with the ROS production of Fe–Zn alloys being lower than that of pure Fe. High concentrations of ROS exhibited apparent toxic effects on ECs and promoted DNA damage. As an antioxidant, Zn^2+^ effectively reduced ROS production around Fe and improved the cell viability on its surface at a concentration of 0.04 mmol/l. This study presents a novel approach to address the oxidative stress caused by Fe implant materials and demonstrates the potential of Fe–Zn alloy as a biodegradable implant material.

## Supplementary Material

rbae002_Supplementary_Data
