# Supplementary material for: Fe–Zn alloy, a new biodegradable material capable of reducing ROS and inhibiting oxidative stress
Source: Regen Biomater. 2024 Jan 12;11:rbae002. doi: 10.1093/rb/rbae002 (PMC10884730; doi:10.1093/rb/rbae002)
Supplement: rbae002_Supplementary_Data [file rbae002_supplementary_data.docx]

***Supplementary data***

**Fe-Zn alloy, a new biodegradable material capable of reducing ROS and inhibiting oxidative stress**

Shuaikang Yang ^a^, Weiqiang Wang ^a*^, Yanan Xu ^a^, Yonghui Yuan ^b*^, Shengzhi Hao ^a^

a School of Materials Science and Engineering, Dalian University of Technology, Dalian 116024, PR China

b Clinical Research Center for Malignant Tumor of Liaoning Province, Cancer Hospital of Dalian University of Technology, Shenyang 110042, PR China

*Correspondence: [wangwq@dlut.edu.cn](mailto:wangwq@dlut.edu.cn) (Weiqiang Wang), [yyxhdh@126.com](mailto:yyxhdh@126.com) (Yonghui Yuan)

**1 Materials and methods**

*1.1 Material Preparation*

A bipolar pulse power (1501DM, SOYI, China) was selected for electrodeposition. The formulation of the electrolyte is shown in Table S1. During deposition, the pH of the electrolyte was controlled at 2.3 by HCl and NaOH, and the temperature was kept at 58 ℃. All chemicals were of analytical grade and were purchased from Shanghai Aladdin Biochemical Technology Co., Ltd (Shanghai, China).

Table S1 Chemical composition of the electrodeposition bath.

| Chemicals | Concentration (g⋅L^−1^) |
| --- | --- |
| FeCl_2_·4H_2_O | 253.49 |
| ZnCl_2_ | 30.67 |
| NaCl | 45.00 |
| MnCl_2_·4H_2_O | 19.79 |
| C_6_H_8_O_6_ | 5.00 |
| C_6_H_5_Na_3_O_7·_2H_2_O | 4.41 |
| C_6_H_4_SO_2_NNaCO·2H_2_O | 1.90 |
| CH_3_(CH_2_)_11_OSO_3_Na | 0.10 |
| H_3_BO_3_ | 30.00 |
| C_3_H_4_O_4_ | 5.20 |

*1.2* *Microstructure characterization*

The crystal phase of the alloys was analyzed by an X-ray diffractometer (XRD, Empyrean, PANalytical B·V, Netherlands) using a Cu Kα irradiation (λ=1.54056 A˚) at 40 kV with a step size of 0.04° and a scan range of 20–100° (in 2θ).

*1.3 Immersion tests*

A static immersion test, according to NACE/ASTM G31-12a^[1]^, was carried out to analyze the corrosion rate of the alloys. The alloys were immersed in Hank’s solution for 7, 28, and 56 d, respectively. The solution was refreshed every 24 hours. After the immersion test, according to ASTM G1-03(2017)e1^[2]^, the loose corrosion products were removed using a soft brush and then cleaned with ultrasonic in distilled water two times. Chemical cleaning was performed in 200 g/L of (NH_4_)_2_HC_6_H_5_O_7_ solution at 75°C for 20 min. At last, these alloys were ultrasonically cleaned in deionized water and absolute ethyl alcohol in sequence and dried. The corrosion rates were calculated by a weight-loss method. Three parallel groups were set up for each type of sample in the immersion test. Finally, the corroded morphology was analyzed by SEM.

$$\text{γ}=\frac{\text{M}_{0}-\text{M}_{1}}{\text{S}\times\text{T}}$$

Where *γ* is the corrosion rate; *M_0_* is the mass of the sample before immersion; *M_1_* is the mass of the sample after immersion; *S* is the exposed surface area of the sample; *T* is the immersion days.

*1.4.* *Cytocompatibility test*

*1.4.1. Cytotoxicity assay*

For the ionic solutions test, the cell suspension was seeded into 96-well plates at a density of 5× 10^4^ cells/mL and 100 μL per well. After the cells adhered to the well, the complete medium was removed and the well was washed with phosphate buffer (PBS, HyClone, USA). Subsequently, 100 μL of ionic solution was added to each well. After incubation for 24 hours and 48 hours, the ionic solution was replaced with 100 μL of complete medium containing 10 μL CCK-8 (Beyotime Biotechnology, China), and the culture was continued for 2 hours. The complete medium containing CCK-8 and cells was used as a positive control, and the complete medium containing CCK-8 but without cells acted as a negative control. The absorbance (OD values) was detected by a microplate reader (SpectraMax M2e, Molecular Devices, USA) at 450 nm. The cell viability was measured by the following equation:

*Viability % = (OD_samples_ - OD_negative_) / (OD_positive_ - OD_negative_) ×* *100%*

The results were based on five paralleled experiments.

*1.4.2. Cell migration assay*

For cell migration assay, the cell suspension was cultured in 6-well plates at a density of 6 × 10^5^ cells/mL and 1 mL per well. After incubating the cells to confluence, a cell-free wound zone was scraped out with a sterile tip and the exfoliated cells were removed with PBS. Then the culture media (ionic solutions) with different ion concentrations were added. The medium without FBS, ECGF, and metal ions added artificially was set as a control group. After incubation for 12 and 24 hours, photographs were taken with a fluorescent microscope (IX83, Olympus, Japan). The results were based on three paralleled experiments.

*1.4.3. Cell adhesion morphology*

The sterilized alloys were placed in 24-well plates and incubated in a cell suspension at a density of 2×10^4^ cells/mL, 1 mL per well. After the cells adhered to the sample for 2, 4, and 8 hours, they were fixed using 4% paraformaldehyde for 10 minutes and stained with Actin-Tracker Green-488 (Beyotime Biotechnology, China) and 4′, 6-diamidino-2-phenylindole (DAPI, Beyotime Biotechnology, China), and then observed by fluorescence microscope.

After the cells were allowed to adhere to the sample for a duration of 24 hours, they were fixed using a 4% paraformaldehyde solution at 4 °C for a period of 2 hours. Subsequently, dehydration was carried out using an ethanol-water solution with varying concentrations (50%, 60%, 70%, 80%, 90%, and 100%). Finally, the samples were air-dried and coated with gold via sputtering in order to facilitate the observation of cell morphology through scanning electron microscopy (SEM).

*1.4.4. Cell proliferation*

For the direct culture, the sterilized alloys were placed in 24-well plates. The cell suspension was added into the 24-well plates with alloys at a density of 5×10^4^ cells/mL and 1 mL per well. After incubation for 1, 3, and 5 days, the alloys were moved into new wells and washed with PBS twice. Subsequently, the medium containing 10% CCK-8 was added and the incubation was continued for 2 hours. Finally, the supernatant was taken into 96-well plates to test the absorbance by the microplate reader at 450 nm.

**2 Supplementary Results**


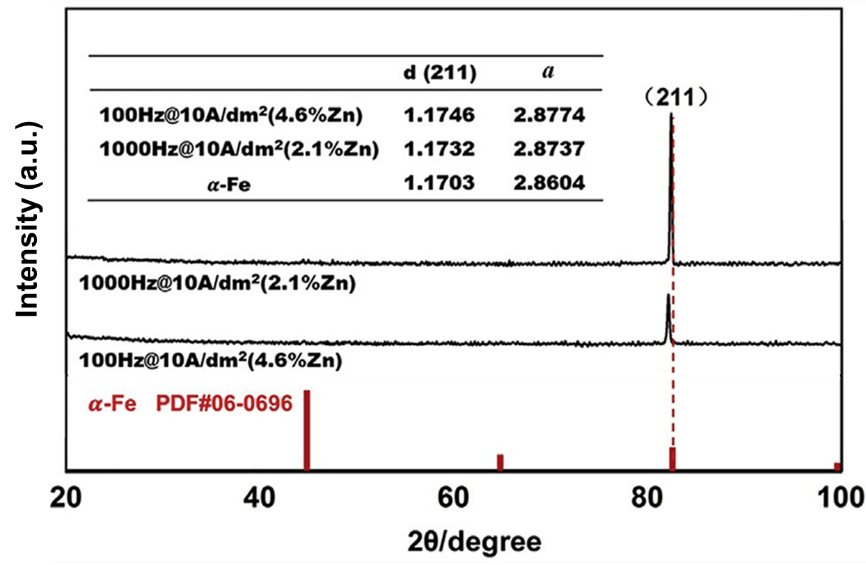


**Figure S1.** XRD patterns of Fe-Zn alloys.


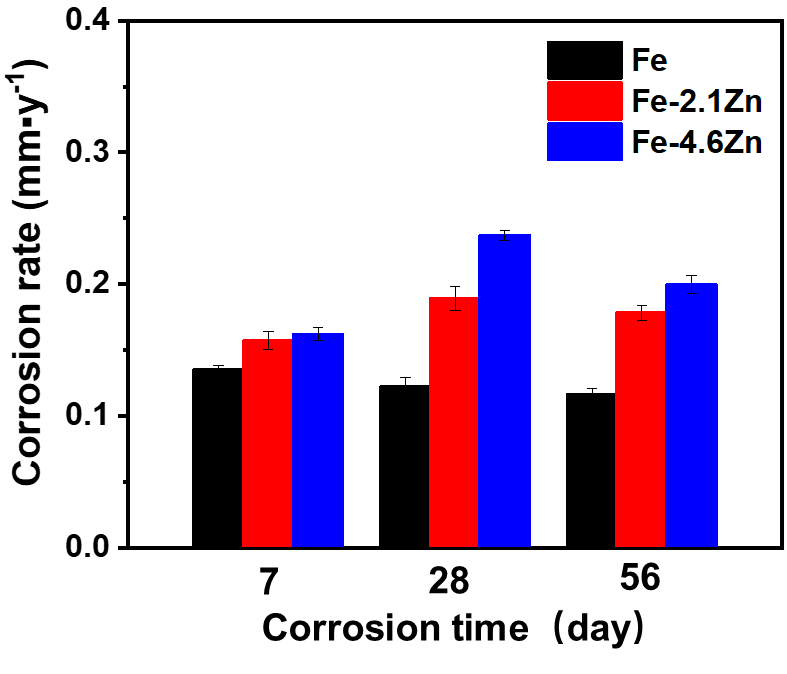


**Figure S2**. Corrosion rates of alloys based on weight loss after static immersion tests.


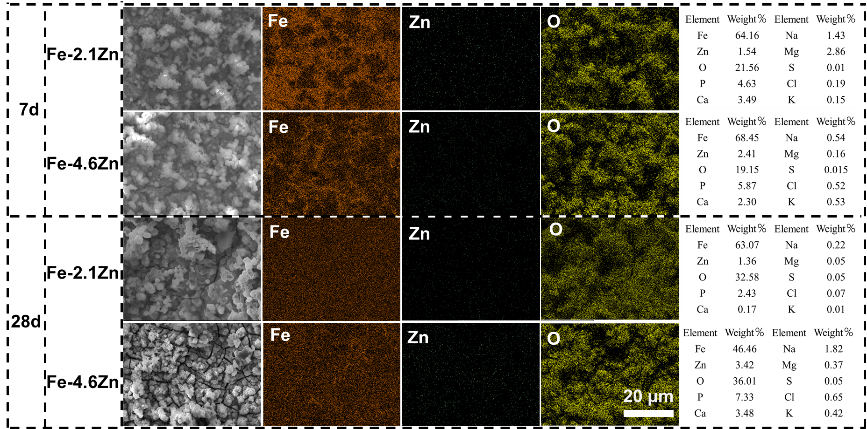


**Figure S3.** Surface morphology and element composition of corrosion products of different alloys after static immersion tests.

**Supplementary references**

[1] NACE/ASTM G31-12aStandard Guide for Laboratory Immersion Corrosion Testing of Metals, ASTM International, West Conshohocken, PA, 2012.

[2] ASTM G1-03 e1, Standard Practice for Preparing, Cleaning, and Evaluating Corrosion Test Specimens, 2017, ASTM International, West Conshohocken, PA, 2017.
